# Clinical utility of cerebrospinal fluid vitamin D-binding protein as a novel biomarker for the diagnosis of viral and bacterial CNS infections

**DOI:** 10.1186/s12879-021-05924-z

**Published:** 2021-03-05

**Authors:** Young Jin Kim, Hương Giang Lê, Byoung-Kuk Na, Bo Gyu Kim, Youn-Kwan Jung, Mutbyul Kim, Heeyoung Kang, Min-Chul Cho

**Affiliations:** 1 Department of Laboratory Medicine, Kyung Hee University Hospital, Kyung Hee University School of Medicine, Seoul, 02447 Republic of Korea; 2 Department of Parasitology and Tropical Medicine, Gyeongsang National University College of Medicine, Jinju, Republic of Korea; 3 BK21Plus Team for Anti-aging Biotechnology and Industry, Department of Convergence Medical Science, Gyeongsang National University, Jinju, Republic of Korea; 4 Biomedial Research Institute, Gyeongsang National University Hospital, Jinju, Republic of Korea; 5 Department of Laboratory Medicine, Gyeongsang National University Hospital, Gyeongsang National University College of Medicine, Jinju, Republic of Korea; 6 Department of Neurology, Gyeongsang National University Hospital, Gyeongsang National University College of Medicine, Jinju, Republic of Korea; 7 Institute of Health Science, Gyeongsang National University, Jinju, Republic of Korea

**Keywords:** Vitamin D-binding protein, Cerebrospinal fluid, Infection, Diagnosis, Biomarker, *GC* genotype

## Abstract

**Background:**

Rapid and accurate diagnosis of central nervous system (CNS) infections is important, and laboratory tests help diagnose CNS infections. Even when the patient has symptoms, laboratory tests often do not reveal any specific findings. The potential of vitamin D-binding protein (VDBP) to be used as a biomarker for viral and bacterial CNS infections was studied.

**Methods:**

A total of 302 subjects with suspected CNS infection who underwent lumbar puncture were included. Clinical and laboratory data were collected retrospectively. VDBP levels were measured in the cerebrospinal fluid (CSF) samples. Genotyping for the *GC* gene encoding VDBP was also performed. VDBP levels were analyzed and compared by CNS infection, pathogen, CSF opening pressure, and *GC* genotype.

**Results:**

A CNS infection group (*n* = 90) and a non-CNS infection group (*n* = 212) were studied. In terms of its receiver operating characteristic, CSF VDBP showed an area under the curve of 0.726 for the diagnosis of CNS infection. CSF VDBP levels were significantly different between the CNS infection and non-infection groups. The CNS infection group with enterovirus showed a statistically lower distribution of CSF VDBP levels than the other virus groups. The group with CSF opening pressure > 25 cmH_2_O showed higher CSF VDBP levels than the other groups. There was no significant difference in *GC* gene allele distribution between the CNS infection and non-infection groups.

**Conclusions:**

CSF VDBP levels were increased in patients with CNS infection. The CSF VDBP showed potential as a new biomarker for viral and bacterial CNS infections.

## Background

The central nervous system (CNS) can be infected with numerous infectious agents, which results in significant morbidity and mortality, despite the effectiveness of available antimicrobials [1–3]. Globally, there were 340,000 deaths estimated by the World Health Organization to be related to meningitis, with an incidence of 700,000. CNS infection involves meningitis, encephalitis, and brain abscess, but the most frequent CNS infection is meningitis. Many infectious pathogens are known to cause CNS infections, including broad categories of bacteria, viruses, fungi, mycobacteria, and parasites [4]. Due to the high morbidity and mortality of CNS infections, rapid diagnosis aimed at providing prompt and appropriate treatment is a priority [5].

The gold standard for the diagnosis of CNS infections is microbiological culture. However, this method shows low sensitivity, and the diagnosis may be delayed [6]. Moreover, in microbiological culture, bacterial growth requires at least 2 days. In cases of viral meningitis, culture may take even longer or may not be feasible. Currently, the most common bacterial and viral pathogens can be detected with high sensitivity by multiplex real-time polymerase chain reaction (PCR), reducing the laboratory turnaround time. However, the disadvantages of this PCR-based molecular test are that only a limited number of pathogens can be detected in advance, and skilled technicians and expensive molecular test equipment are required.

In clinical situations, the initial diagnosis of CNS infection, including meningitis, usually relies on the cytological and biochemical characteristics of cerebrospinal fluid (CSF), culture results, neuroimaging, and clinical manifestations. A lumbar puncture, also referred to as spinal tap, is the commonly performed procedure to obtain CSF to diagnose meningitis [7, 8]. The most rapidly available results from a lumbar puncture include cell counts and total protein, glucose, and lactate concentrations. However, caution is required because CSF cell count is normal in up to 15% of patients with some viral meningitis [9]. Thus, if clinical symptoms suggest a diagnosis of meningitis, there is a need for additional diagnostic biomarkers to identify these cases of viral meningitis despite the absence of pleocytosis [10]. Furthermore, the features of meningitis in CSF analysis may not appear in the elderly or in patients with immunosuppression, and differential cell counts of CSF is subjective and dependent on the examiner [11]. Thus, additional objective biomarkers are necessary for the accurate diagnosis of CNS infections, including meningitis.

Vitamin D-binding protein (VDBP) is a 58-kDa multifunctional protein known to be an acute phase reactant whose level can change depending on various conditions [12–15]. Although VDBP was originally known to play a major role in vitamin D metabolic transport, various other roles of VDBP have been reported, including extracellular actin sequestration and activation of the complement system [16]. The gene encoding VDBP (*GC*) has a high rate of polymorphism. Two single-nucleotide polymorphisms (SNPs), rs7041 and rs4588, have given rise to three major polymorphic isoforms of VDBP (GC1F, GC1S, and GC2). Their frequencies also differ among ethnic populations [17]. The level of VDBP expression is relatively lower in the various body fluids, including CSF, compared with its level in blood. Although VDBP has been detected in human CSF, it is not yet clear whether VDBP is synthesized directly in the central nervous system or transported from general circulation through the blood brain barrier.

VDBP is known to potentially modulate the inflammatory and immune responses in the blood and body fluids [16]. In a previous study, increased VDBP levels in cervicovaginal fluid could be used as a non-invasive biomarker of intraamniotic infection and impending preterm delivery in women with the impending infections [18]. Studies on VDBP in CSF have been mainly conducted on neurodegenerative diseases, such as multiple sclerosis and Alzheimer’s disease, however there are very few studies on VDBP concentrations during CNS infections [19]. Thus, the main purpose of the present study was to evaluate the clinical utility of CSF VDBP as a novel biomarker for the diagnosis of viral and bacterial CNS infections in a relatively larger number of patients. In addition, we tried to determine whether the concentrations of CSF VDBP differ depending on either the severity of the CNS infection or the causative pathogen.

## Methods

### Study subjects

This prospective study enrolled 302 patients who underwent lumbar puncture for the purpose of diagnosis from September 2017 to May 2020. We collected clinical and laboratory data including age, sex, cellular differential counts of CSF, chemical analysis of CSF (including glucose and protein levels), and the final diagnosis from electronic medical records (EMR). Culture and molecular laboratory tests, including multiplex PCR (Seeplex Meningitis-V1 ACE Detection kit and Seeplex Meningitis-B ACE Detection [Seegene, Seoul, Korea]) and FilmArray (BioFire Diagnostics, LLC, Salt Lake City, UT) Meningitis/Encephalitis ME panel were utilized at the clinicians’ discretion for the identification of pathogens. The remaining CSF samples were stored at − 80 °C until the CSF VDBP measurements.

These enrolled patients were classified into two groups (CNS infection and non-CNS infection) depending on the results of CSF analysis and the final diagnosis. We classified patients as having CNS infections if the results of CSF analysis, imaging studies, or clinical symptoms strongly suggested meningitis, meningoencephalitis, or encephalitis. The differential diagnosis for meningitis, encephalitis, and meningoencephalitis is mainly made by clinical symptoms, and the criteria are as follows [20]. Meningitis is an infection only in the meninges, not the brain parenchyma; therefore, the clinical symptoms of the patient were mostly meningeal irritation signs, such as headache and neck stiffness, without neurologic symptoms such as decreased consciousness or focal neurologic deficits. Encephalitis is an infection caused in the brain parenchyma, not in the meninges; thus, the patient’s clinical symptoms were mostly neurologic, such as decreased consciousness or seizure, but signs of meningeal irritation were not observed. Meningoencephalitis is an infection of both the meninges and brain parenchyma; therefore, patients complained of both neurologic symptoms and signs of meningeal irritation. The CNS infection group included cases in which viruses or bacteria were detected in the CSF. All other non-infectious etiologies, such as autoimmune meningitis, acute phase of multiple sclerosis/ neuromyelitis optica, and cancer-related meningitis were excluded by related laboratory tests or image workups and chart reviews. If the result of CSF analysis was normal and the final diagnosis was not related to CNS infection, patients were classified into the non-CNS infection group.

When the number of red blood cells exceeded 1000 per mm^3^ in differential counts for CSF, it was regarded as traumatic tapping and excluded from this study. With reference to a previous study [21], 25 cmH2O was considered to be the cut off for high opening pressure. Thus, among the patients with CNS infection, patients with a record of opening pressure were divided into two groups (> 25 cmH_2_O and ≤ 25 cmH_2_O). The distributions of VDBP in the two groups were compared.

### CSF VDBP measurements

CSF VDBP levels were measured using an enzyme-linked immunosorbent assay kit (R&D Systems, Minneapolis, MN, USA) according to the manufacturer’s protocol.

### *GC* gene genotyping

Genomic DNA was isolated from peripheral blood leukocytes using a DNeasy Blood and Tissue Kit (Qiagen, Hilden, Germany) according to the manufacturer’s instructions. *GC* gene genotyping for rs7041 and rs4588 was performed using a TaqMan SNP Genotyping Assay (Thermo Fisher Scientific, Waltham, MA, USA) and an ABI ViiA 7 Real-Time PCR System (Applied Biosystems, Foster City, CA, USA) according to the manufacturer’s instructions. *GC* gene alleles were determined as follows: Gc1f (c.1296 T; c.1307C), Gc1s (c.1296G; c.1307C), and Gc2 (c.1296 T; c.1307A). In addition, the distribution of CSF VDBP levels according to *GC* allele was also compared in the CNS infection group and non-CNS infection group, respectively. For simplicity of comparison, only CSF VDBP levels from subjects with homozygous *GC* alleles were used.

### Statistical analysis

T-tests or Mann-Whitney U tests were used to compare variables depending on the normality of CSF VDBP distribution. Pearson’s Chi-square test was used to compare the proportion of categorical variables. Box-and-whisker plots were used to demonstrate the VDBP distribution. Receiver operating characteristic (ROC) curve analysis was used to evaluate the diagnostic value of CSF VDBP for CNS infection. The Kruskal-Wallis test was used to test for differences in the VDBP distribution by *GC* genotype and pathogen. All statistical analyses were performed using MedCalc Statistical software, version 17.2 (Mariakerke, Belgium). *P* values < 0.05 were considered statistically significant.

## Results

### General characteristics of study subjects and the results of CSF analysis

A total of 302 patients were enrolled in this study. The median age of the study subjects was 31, and the number of patients under 18 years old was 129. A total of 166 male subjects were included, and they comprised 55.0% of the subjects. Among them, 90 (29.8%) and 212 (70.2%) patients were classified in the CNS infection group and non-CNS infection group, respectively, based on the final diagnosis in the electronic medical records. There were no significant differences in median age, the number of patients under 18 years old, or the number of males between the two groups. In the CSF analysis, the CNS infection group showed higher total protein levels, WBC counts, and VDBP levels than the non-CNS infection group. The general characteristics of the study subjects and the results of the CSF analysis are shown in Table 1.

**Table 1:** Characteristics of included patients and CSF analysis

	All patients (*n* = 302)	CNS infection (n = 90)	non-CNS infection (*n* = 212)	*P*
Age (Yr)	31 (1.9–63.0)	25.5 (6.0–55.0)	37.5 (0.3–65.0)	0.950
Patients age < 18	129 (42.72)	38 (42.22)	91 (42.92)	0.924
Males	166 (54.97)	39 (43.33)	127 (59.91)	0.144
CSF analysis				
Total protein (g/L)	0.38 (0.30–0.60)	0.64 (0.36–1.05)	0.34 (0.25–0.48)	< 0.001
Glucose (mg/dL)	65.0 (58.0–750.)	63.0 (55.0–74.0)	65.5 (59.0–75.0)	0.063
WBC count (× 109/L)	0 (0–0.02)	0.09 (0.02–0.27)	0 (0–0)	< 0.001
VDBP (μg/mL)	1.00 (1.00–2.00)	3.20 (1.70–5.10)	1.10 (0.80–1.80)	< 0.001

*Abbreviations*: *CNS* central nervous system, *AST* Aspartate Aminotransferase, *ALT* alanine transaminase, *CRP* C-reactive protein, *CSF* cerebrospinal fluid, *WBC* white blood cells VDBP, vitamin D-binding protein

Data are presented as medians (interquartile range) or numbers (percentages)

### Etiology of CNS infection

Among the 90 patients with CNS infections, 80 were diagnosed with meningitis, 8 with encephalitis, and 2 with meningoencephalitis. Of the 80 patients with meningitis, 41 patients had pathogens detected in laboratory tests, including molecular tests. These pathogens included Enterovirus (*n* = 29), Epstein-Barr virus (EBV) (*n* = 3), varicella zoster virus (VZV)(*n* = 4), herpes simplex virus type 2 (HSV2) (*n* = 1), human herpesvirus 6 (HHV6)(n = 1), and EBV and VZV detected simultaneously (*n* = 1), whereas *Neisseria meningitidis* (n = 2) was detected by culture. The remaining 39 patients were clinically diagnosed based on an imaging study, CSF cell counts, and clinical findings checked by neurology physicians. Among the eight encephalitis patients, one patient had a pathogen detected in a molecular test (EBV, n = 1), and no pathogen was detected in the other seven patients. Among the two meningoencephalitis patients, one patient had a pathogen detected in a molecular test (EBV, n = 1), and no pathogen was detected in the other patient. The etiologies of the CNS infections are summarized in Table 2.

**Table 2:** Etiologies of CNS infections

Disease and pathogen	CNS infection (n = 90)
Meningitis	80
Enterovirus	29
EBV	3
VZV	4
HSV2	1
HHV6	1
EBV + VZV	1
*Neisseria meningitidis*	2
Non-identified	39
Encephalitis	8
EBV	1
Non-identified	7
Meningoencephalitis	2
EBV	1
Non-identified	1

*Abbreviations*: *CNS* central nervous system, *EBV* Epstein-Barr virus, *VZV* varicella zoster virus, *HSV2* herpes simplex virus type 2, *HHV6* human herpesvirus 6

### Comparison of CSF VDBP levels between the CNS infection and non-CNS infection groups

**Fig. 1:**
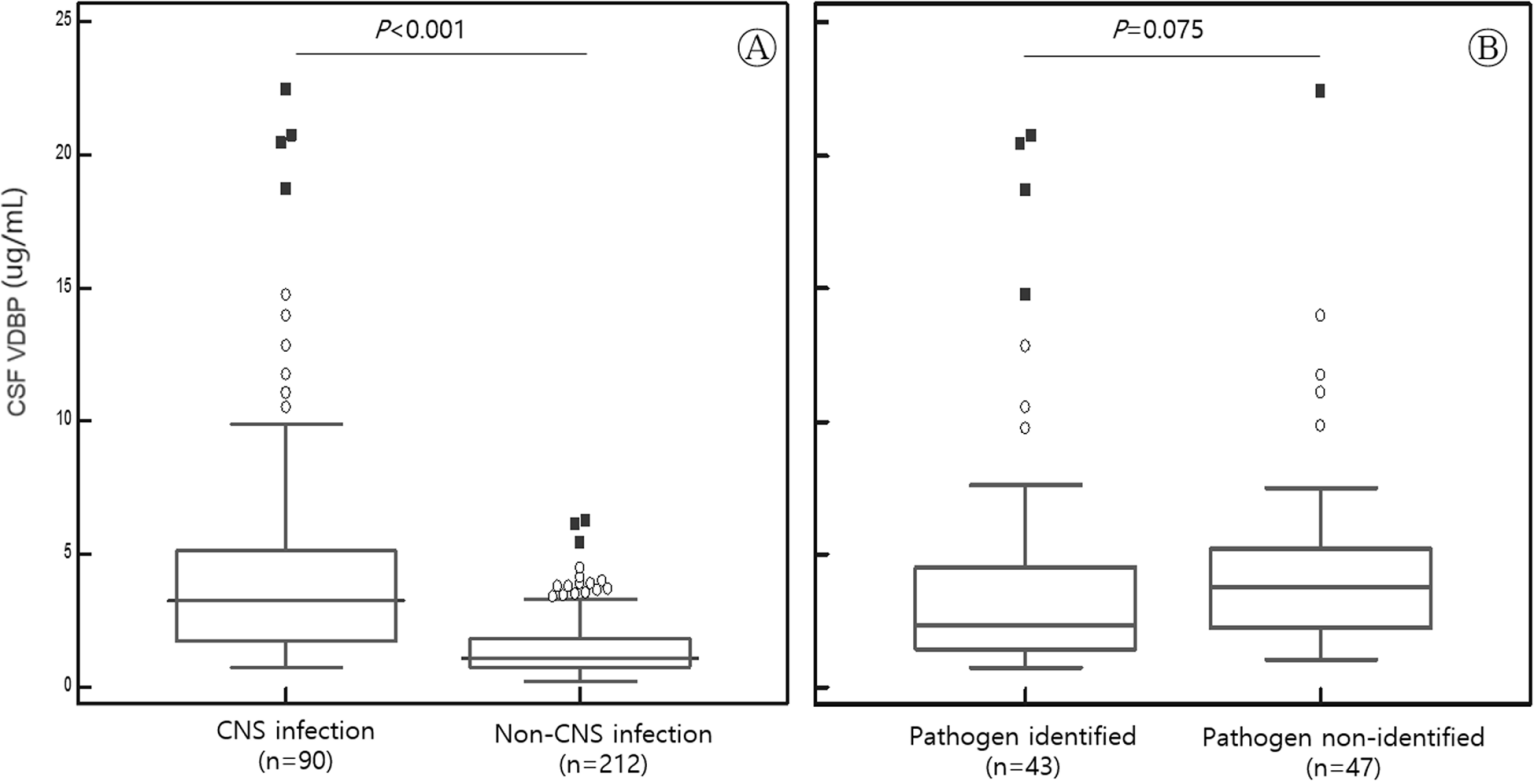
Comparison of median CSF VDBP levels and interquartile ranges (**a**) The difference in CSF VDBP levels between the CNS infection (3.20 μg/mL, 1.70–5.10) and non-CNS infection groups (1.10 μg/mL, 0.80–1.80)(*P* < 0.001). (**b**) The difference in CSF VDBP levels between the pathogen-identified group (2.34 μg/mL, 1.46–4.53) and non-identified group (3.76 μg/mL, 2.25–5.21)(*P* = 0.075) within the CNS infection group. Outliers of 1.5 to 3 IQR are marked with circles, and outliers > 3 IQR are marked with squares. Abbreviations: VDBP, vitamin D-binding protein; CNS, central nervous system; IQR, interquartile range

CSF VDBP levels in the study subjects are summarized in Table 1. The median (interquartile range [IQR]) CSF VDBP level in all patients was 1.00 (1.00–2.00) μg/mL. The median (IQR) CSF VDBP level in the CNS infection group (*n* = 90) and non-CNS infection group (*n* = 212) was 3.20 (1.70–5.10) μg/mL and 1.10 (0.80–1.80) μg/mL, respectively. CSF VDBP levels in the CNS infection group were significantly higher than those in the non-CNS infection group (*P* < 0.001) (Fig. 1a). Among the 90 patients in the CNS infection group, the median (IQR) CSF VDBP level in the pathogen-identified group (*n* = 43) and the pathogen-non-identified group (*n* = 47) was 2.34 μg/mL (1.46–4.53) and 3.76 μg/mL (2.25–5.21), respectively, and the difference between the groups was not significant (*P* = 0.075) (Fig. 1b). The median (IQR) CSF VDBP level was also compared between the CNS infection group with normal CSF cell counts (*n* = 7, 1.8 μg/mL [1.19–2.94]) and CNS infection group with pleocytosis in the CSF cell count (*n* = 83, 3.46 μg/mL [1.80–5.38]). The difference in CSF VDBP levels between the two groups was statistically significant (*P* = 0.035).

### Receiver operating characteristic analysis for the diagnosis of CNS infection using CSF VDBP level

**Fig. 2:**
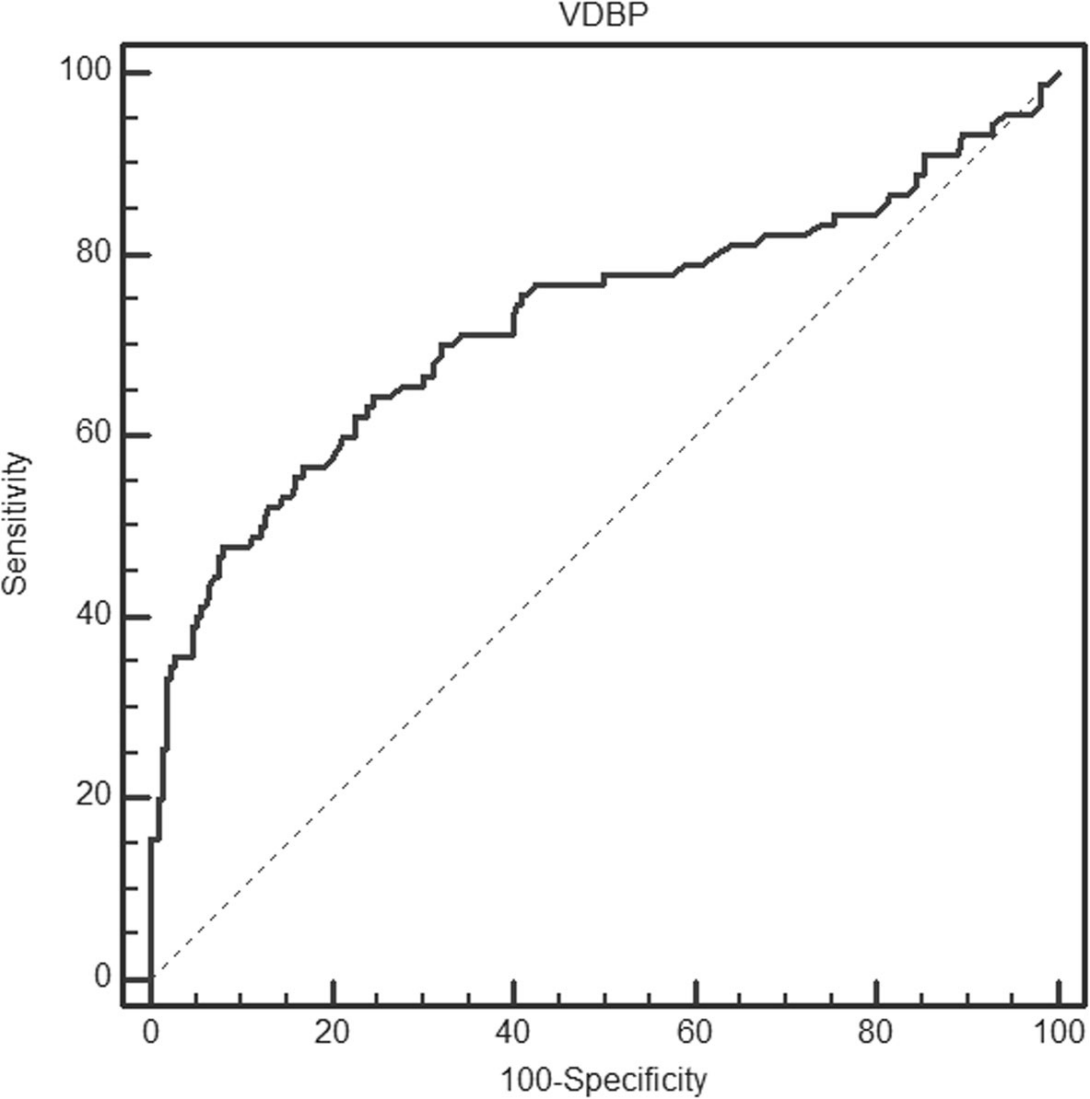
ROC curve for VDBP versus CNS infections showed an AUC of 0.726. CNS infections included both clinically diagnosed cases and laboratory-confirmed cases

We performed ROC analysis for the detection of CNS infection using the CSF VDBP level. The ROC curve analysis of VDBP for diagnosing CNS infections showed an area under the curve (AUC) of 0.726 (95% confidence interval = 0.672–0.776, *P* < 0.001)(Fig. 2), and the cutoff value of VDBP for maximum sensitivity (64.44%) and specificity (75.47%) was 1.37 μg/mL (Fig. 2).

### Comparison of CSF VDBP levels according to the pathogens in CNS infection patients

**Fig. 3:**
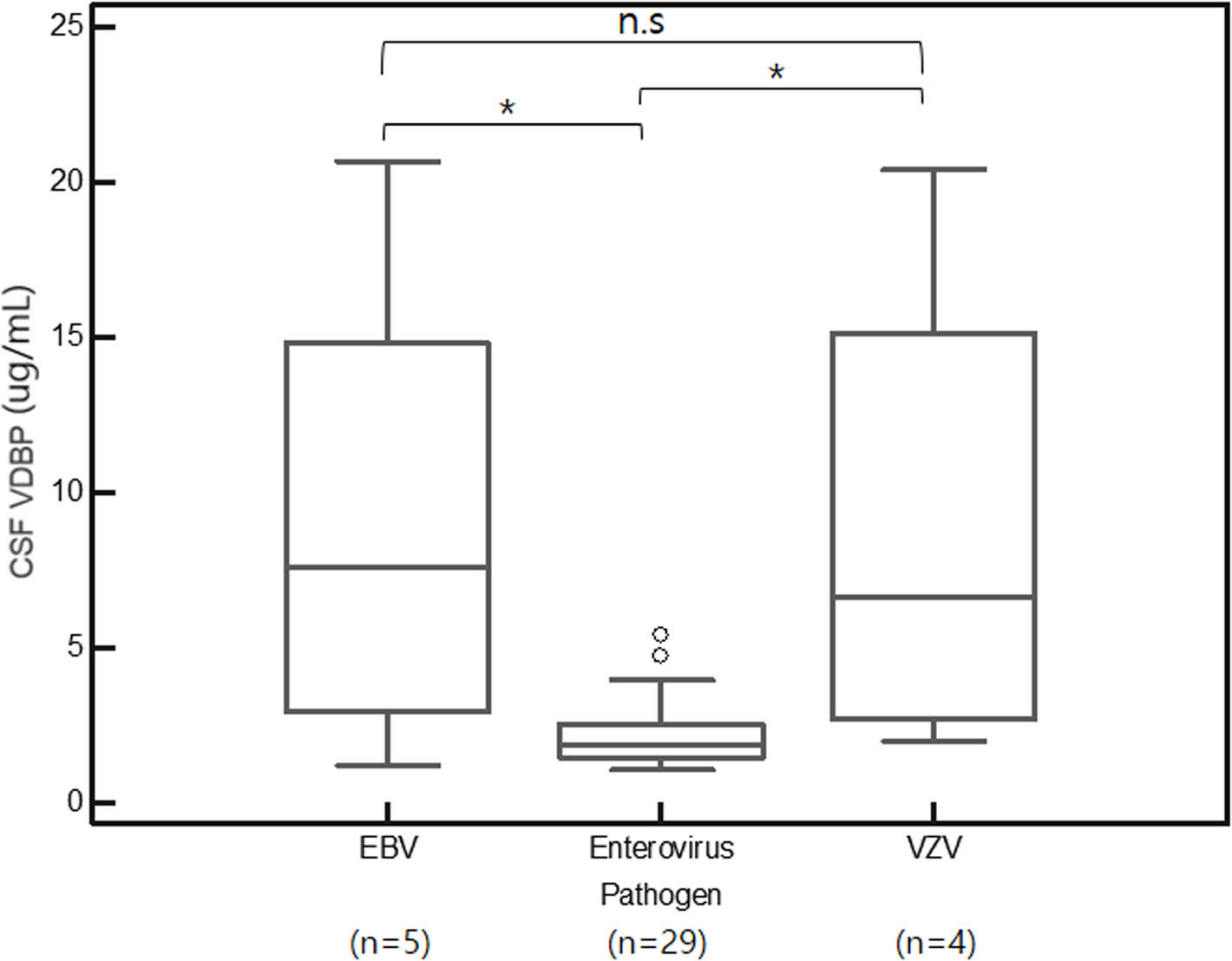
CSF VDBP level comparisons among the EBV (n = 5), enterovirus (n = 29), and VZV (n = 4) groups. The Enterovirus group (median, IQR; 1.89 μg/mL, 1.44–2.54) showed significantly lower distribution than the EBV (7.58 μg/mL, 2.93–14.79) and VZV (6.62 μg/mL, 2.72–15.10) (*P* = 0.022) groups. Outliers of 1.5 to 3 IQR are marked with circles, and outliers > 3 IQR are marked with squares. * Statistically significant difference (*P* < 0.05) according to the Kruskal-Wallis test; n.s.: not significant. Abbreviations: VDBP, vitamin D-binding protein; CSF, cerebrospinal fluid; EBV, Epstein-Barr virus; ZVZ, varicella zoster virus

We compared the CSF VDBP levels in the CNS infection group to determine whether CSF VDBP levels were different depending on the pathogens of CNS infection. In the comparison of the distribution of CSF VDBP levels among infections caused by pathogens that were identified at least three times (EBV [*n* = 5], enterovirus [*n* = 29], and VZV [*n* = 4]), the enterovirus group (median, IQR; 1.89 μg/mL, 1.44–2.54) showed a significantly lower distribution of CSF VDBP levels than the EBV (7.58 μg/mL, 2.93–14.79) and VZV (6.62 μg/mL, 2.72–15.10) groups (*P* = 0.022) (Fig. 3). The EBV and VZV groups were not significantly different from each other (*P* = 0.807). *Neisseria meningitidis* was identified in two cases, and the CSF VDBP levels in those two cases were 10.53 μg/mL and 14.74 μg/mL, respectively. The CSF VDBP levels of other pathogens identified in only one case are as follows: HSV2 (18.69 μg/mL), HHV6 (0.76 μg/mL), and simultaneous detection of EBV and VZV (6.67 μg/mL).

### Correlation between CSF VDBP levels and the CSF opening pressure

**Fig. 4:**
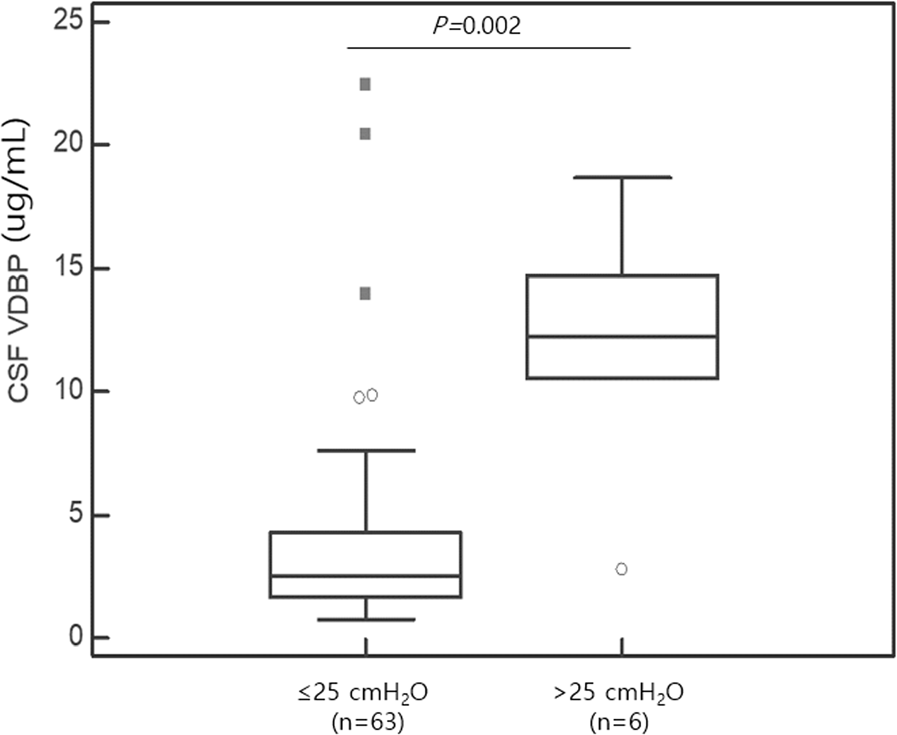
The CSF VDBP level distribution according to CSF opening pressure. The VDBP level shows significant differences between patients with ≤25 cmH_2_O pressure (median, IQR; 2.49 μg/mL, 1.64–4.28) and those with pressures > 25 cmH_2_O (12.29 μg/mL, 10.53–14.74) within the CNS infection group (*P* = 0.002). Outliers of 1.5 to 3 IQR are marked with circles and outliers > 3 IQR are marked with squares. Abbreviations: VDBP, vitamin D-binding protein; CSF, cerebrospinal fluid; CNS, central nervous system; IQR, interquartile range

There were 69 patients with a record of opening pressure at the time of cerebrospinal fluid puncture in the CNS infection group. Among them, 6 patients showed CSF opening pressures > 25 cmH_2_O, and the remaining 63 patients showed pressures ≤25 cmH_2_O. Among the patients with an opening pressure > 25 cmH_2_O, two had *Neisseria meningitidis*, one had EBV, one had HSV2, and two patients had non-identified pathogens. They showed significantly higher CSF VDBP levels than those with an opening pressure ≤ 25 cmH_2_O (*P* = 0.002) (Fig. 4).

### Genotype and allele frequencies of the *GC* gene encoding VDBP

Gc1s-1f (*n* = 82, 27.15%) was the most common genotype, followed by Gc2-1f type (*n* = 69, 22.85%) and Gc1f-1f type (*n* = 57, 18.87%) among all study patients. The three most frequent genotypes were in the order Gc1f-1f > Gc1s-1f > Gc2-1f in the CNS infection group, and Gc1s-1f > Gc2-1f > Gc1f-1f in the non-CNS infection group. There was no significant difference in genotype distribution between the two groups (*P* = 0.686, Table 3). Gc1f (*n* = 265, 43.87%) was the most common allele, followed by Gc2 (*n* = 174, 28.81%) and Gc1s (*n* = 165, 27.32%) among all study patients. The most frequent alleles were in the order of Gc1f > Gc2 > Gc1s in the CNS infection group, and Gc1f > Gc1s > Gc2 in the non-CNS infection group. There was no significant difference in allele distribution between the two groups (*P* = 0.913, Table 3). For comparison of CSF VDBP levels by *GC* allele, there were 41 subjects in the CNS infection group and 72 subjects in the non-CNS infection group with homozygous *GC* alleles. The distribution of VDBP was not significantly different among the *GC* allele types in either the CNS infection group (*P* = 0.561, Kruskal–Wallis test) or the non-CNS infection group (*P* = 0.135).

**Table 3:** *GC* genotype and allele frequencies

	All patients (n = 302)	CNS infection (n = 90)	non-CNS infection (n = 212)	*P*
*GC* genotype				0.686
Gc1f-1f	57 (18.87)	22 (24.44)	35 (16.51)	
Gc1s-1f	82 (27.15)	19 (21.11)	63 (29.72)	
Gc1s–1 s	25 (8.28)	7 (7.78)	18 (8.49)	
Gc2-1f	69 (22.85)	16 (17.78)	53 (25.00)	
Gc2–1 s	33 (10.93)	12 (13.33)	21 (9.91)	
Gc2–2	36 (11.92)	14 (15.56)	22 (10.38)	
*GC* allele				0.913
Gc1f	265 (43.87)	79 (43.89)	186 (43.87)	
Gc1s	165 (27.32)	45 (25.00)	120 (28.30)	
Gc2	174 (28.81)	56 (31.11)	118 (27.83)	

Data are presented as numbers (percentages)

## Discussion

Infections of the CNS often lead to significant morbidity and mortality if not recognized and treated promptly. Therefore, it is imperative for appropriate therapy to be initiated quickly when CNS infection is suspected. For early diagnosis, rapid molecular tests for detection of common pathogens are applied in the clinical field and aid in diagnosis [22]. However, the pathogens remain unidentified in many cases of CNS infection [23]. To overcome this, new biomarkers that could help diagnose CNS infection are needed.

In our study, we found that the concentration of CSF VDBP increased significantly in the CNS infection group compared to the non-CNS infection group. Furthermore, ROC curve analysis showed a relatively good predictive value for CSF VDBP levels for the diagnosis of CNS infection with an AUC of 0.726. At a cut-off value of 1.37 μg/mL, CSF VDBP for the diagnosis of CNS infection showed a sensitivity of 64.22% and a specificity of 85.9%. This finding implies that the CSF VDBP level could be considered a new biomarker to diagnose CNS infections. However, this does not mean that CSF VDBP should be the sole biomarker used for diagnosing CNS infections.

Our research team had observed that the CSF VDBP level was significantly increased in patients with meningitis and has reported that CSF VDBP concentration can potentially be considered a new biomarker for the diagnosis of meningitis, in a previous study [19]. However, in that study, only a small number of samples could be analyzed, including those from 102 patients, of which just 17 patients had meningitis. Thus, in the present study, to overcome the limitations of the previous study, a relatively larger number (302) of patients were enrolled, and 90 CNS infection cases were included; among them, in 43 cases, the pathogen involved in the CNS infection was identified. The identified pathogens included various viruses, such as enterovirus, EBV, VZV, HSV2, and HHV6, and two cases involved the bacterium *Neisseria meningitidis*. In addition to meningitis, meningoencephalitis and encephalitis cases were included and analyzed. In this larger-scale study, as in the previous study, CSF VDBP was significantly increased in the CNS infection patient group, including in the CNS meningitis group, reconfirming that CSF VDBP could be a novel biomarker for the diagnosis of viral and bacterial CNS infections. In addition, when we compared CSF VDBP levels between patients with CNS infections and normal CSF cell counts and those with CNS infections with pleocytosis in the CSF cell counts, the CSF VDBP level was significantly higher in the group with pleocytosis. However, since the number of CNS-infected patients with normal CSF cell counts was relatively small (*n* = 7), the potential for CSF VDBP to be used as a single diagnostic marker of CNS infection in the group with normal CSF cell counts could not be determined from our data.

VDBP is a protein that has many functions, including the transport of vitamin D metabolites, actin sequestration, and regulation of immune responses [16]. VDBP in CSF has been mainly studied in neuro-inflammatory and neurodegenerative diseases such as multiple sclerosis. In particular, it has been reported that VDBP concentration in CSF increases and that VDBP scavenges extracellular actin in multiple sclerosis animal models [24]. However, there are very few studies examining the change in VDBP levels during pathogen-invading CNS infections. In addition, VDBP is a protein that is synthesized by the liver and secreted into the general circulation. Although VDBP has been found in human CSF in a previous study [25], it is still not clear how CSF VDBP levels increased in the patients with CNS infections that we observed in this study. Two possibilities might explain the origin of increased CSF VDBP during viral and bacterial CNS infections. First, the tight junction of the blood-brain barrier (BBB) is disrupted, and more VDBP may pass through the BBB to the CSF from the general circulation. This mechanism has been previously reported as inflammation and is intrinsically linked to the mechanisms of tight junction deregulation, which contribute to the loss of BBB in multiple sclerosis animal models [26]. Second, astrocytes and microglial cells, which are intrinsic immune cells in the CNS, might be self-synthesized in response to infection. To confirm these two possibilities, additional studies such as animal model experiments or cell experiments would be necessary.

In the comparison of CSF VDBP levels among cases with identified pathogens (enteroviruses, EBV, and VZV), the enterovirus group showed a lower distribution than the other two groups. Enterovirus is the most common causative virus of meningitis and cases in which aseptic meningitis is caused by either enterovirus, EBV, or VZV are usually self-limited with good prognoses [27]. The immune response and clinical features of enterovirus CNS infections are different from those of other viral pathogens. Enteroviruses cause a more acute onset of meningitis compared to other viral agents [28, 29]. In addition, CSF pleocytosis is absent in approximately 15% of enterovirus infections [9]. Therefore, it can be assumed that the low CSF VDBP level observed in enterovirus infections compared to that in the other pathogen infections in our study might be because the immune response to enterovirus is different from that to the other pathogens. Although the distribution of CSF VDBP showed a significant difference, the number of cases involving other viruses, such as EBV (*n* = 5) and VZV (*n* = 4), were not sufficient to statistically compare with the enterovirus cases; thus, it may be difficult to determine clinical significance and draw conclusions from the current results. Further studies are necessary to elucidate this observation.

The opening pressure during CSF tapping reflects the intracranial pressure (ICP). Meningitis causes increased intracranial pressure (IICP), which leads to clinical symptoms such as headache, nausea, vomiting, and cranial nerve palsy [30, 31]. It is known that if IICP becomes severe, the patient falls into a coma and brain herniation develops, leading to death [31]. Thus, IICP is associated with mortality in meningitis patients and affects the course and prognosis of the disease [30, 32]. Therefore, controlling IICP can improve the prognosis [33, 34]. The opening pressure in meningitis is associated with the severity of the disease, and in our study, the CSF VDBP level was higher in patients with CNS infections with high opening pressure (> 25 cmH_2_O) than in patients with CNS infections with low opening pressure (≤25 cmH_2_O). Therefore, this result suggests that CSF VDBP level might be useful not only for the diagnosis of viral and bacterial CNS infections but also for the assessment of meningitis severity. CSF opening pressure has been reported to increase in bacterial meningitis and approach the reference range in viral meningitis and also to increase due to posture and patient anxiety [35, 36]. In our study, two cases of bacterial pathogens were included in the group with opening pressure > 25 cmH_2_O, and CSF VDBP in this group showed a higher distribution than in the group with opening pressures ≤25 cmH_2_O. Therefore, it will be worth further research to determine whether CSF VDBP could be an objective biomarker that specifically increases in bacterial CNS infections.

The major *GC* genotype and allele frequencies are known to vary among ethnicities [17]. The majority of African American subjects have the Gc1f allele (Gc1f-Gc1f, Gc1f-Gc1s, or Gc1f-Gc2). In contrast, the Gc1f allele is rare in white subjects, but Gc1s-Gc1s and Gc1s-Gc2 are the most frequent in this group [37]. It has been reported that Korean individuals have a different distribution of *GC* allele frequencies than African American and Caucasian individuals [38]. A previous study reported that Gc1f-Gc2 (25%) is the most frequent genotype, followed by Gc1f-Gc1f (22%), Gc1f-Gc1s (20%), and Gc1s-Gc2 (18%) in Korean populations [38], which was consistent with the frequencies observed in our study. As in our previous study [19], the *GC* genotype and allele frequency did not show any statistical correlation with the presence or absence of CNS infections. The *GC* genotype exhibits differences in affinity for vitamin D metabolites with the hierarchy of affinity binding being Gc1f > Gc1s > Gc2 [12]. In addition, experimental evidence has shown that *GC* genotype can influence serum VDBP levels [39, 40]. Furthermore, Gc2 is less able to activate macrophages, resulting in reduced macrophage function in Gc2 carriers compared to carriers of Gc1f and Gc1s in blood [41]. However, in our study, no difference in the CSF VDBP level was observed among the *GC* alleles (*P* = 0.561). This might originate from the difference between CSF and blood, and further studies are needed to evaluate the difference in function and concentration of CSF VDBP among *GC* genotypes.

Our study has several limitations. First, our study did not differentiate between children and adults. The common CNS infection pathogens and the concentration of CSF VDBP may differ by age group, so further research for examining different age groups is needed. Second, in the CNS infection group, not all patients were found to have laboratory-confirmed pathogens. In addition, cases of infection in the non-CNS infection group cannot be ruled out because PCR and culture tests were not performed for all potential pathogens.

## Conclusions

In summary, we reconfirmed that CSF VDBP levels were increased in patients with viral and bacterial CNS infections. We also observed that the CSF VDBP level has the potential to reflect the severity of meningitis. Thus, through this study, we suggest that CSF VDBP has clinical utility as a new biomarker for the diagnosis of viral and bacterial CNS infections.

## Data Availability

The datasets used and analyzed during the current study are available from the corresponding author on reasonable request.

## Abbreviations

AUC: Area under the curve
CNS: Central nervous system
VDBP: Vitamin D-binding protein
CSF: Cerebrospinal fluid
PCR: Polymerase chain reaction
ROC: Receiver operating characteristic
EBV: Epstein-Barr virus
VZV: Varicella zoster virus
HSV2: Herpes simplex virus type 2
HHV6: Human herpesvirus 6
BBB: Blood brain barrier
ICP: Intracranial pressure
IICP: Increased intracranial pressure
